# The Role of Bcl-2 Family Proteins in Therapy Responses of Malignant Astrocytic Gliomas: Bcl2L12 and Beyond

**DOI:** 10.1100/2012/838916

**Published:** 2012-02-14

**Authors:** Fotini M. Kouri, Samuel A. Jensen, Alexander H. Stegh

**Affiliations:** Ken and Ruth Davee Department of Neurology, The Northwestern Brain Tumor Institute, The Robert H. Lurie Comprehensive Cancer Center, Chicago, IL 60611, USA

## Abstract

Glioblastoma (GBM) is a highly aggressive and lethal brain cancer with a median survival of less than two years after diagnosis. Hallmarks of GBM tumors include soaring proliferative indices, high levels of angiogenesis, diffuse invasion into normal brain parenchyma, resistance toward therapy-induced apoptosis, and pseudopallisading necrosis. Despite the recent advances in neurosurgery, radiation therapy, and the development of targeted chemotherapeutic regimes, GBM remains one of the deadliest types of cancer. Particularly, the alkylating agent temozolomide (TMZ) in combination with radiation therapy prolonged patient survival only marginally, and clinical studies assessing efficacies of targeted therapies, foremost ATP mimetics inhibiting the activity of receptor tyrosine kinases (RTKs), revealed only few initial responders; tumor recurrence is nearly universal, and salvage therapies to combat such progression remain ineffective. Consequently, myriad preclinical and clinical studies began to define the molecular mechanisms underlying therapy resistance of GBM tumors, and pointed to the Bcl-2 protein family, in particular the atypical member Bcl2-Like 12 (Bcl2L12), as important regulators of therapy-induced cell death. This review will discuss the multi-faceted *modi operandi* of Bcl-2 family proteins, describe their roles in therapy resistance of malignant glioma, and outline current and future drug development efforts to therapeutically target Bcl-2 proteins.

## 1. Malignant Astrocytic Gliomas—Scope of the Problem

Primary brain and CNS tumors are one of the deadliest human cancers. Based on histological, immunohistochemical, and ultrastructural criteria, the more common astrocytic tumors are graded as pilocytic astrocytoma, grade I; astrocytoma, grade II; anaplastic astrocytoma, grade III; glioblastoma (GBM), grade IV [1]. Patients diagnosed with GBM, the most prevalent and aggressive form of malignant glioma, survive for a mere 12–15 months and suffer from seizures, visual, cognitive, and overall physical decline, memory loss, and personality changes, which drastically impact quality of life [2]. Unfortunately, this dismal prognosis has not changed significantly over the past decades and candidly revealed the major challenges in disease management: (i) the highly aggressive and infiltrating growth of GBM tumors, which almost always prevents complete surgical resection, (ii) the intense resistance of GBM cells toward therapy-induced apoptosis, and (iii) the inefficient penetration of chemotherapeutic drugs across the blood-brain barrier (BBB), and blood-tumor barrier (BTB), toward distal sites of tumor growth. 

Numerous studies focused on defining genomic, genetic, and proteomic aberrations in GBM tumors, which cause or contribute to the phenotypic hallmarks of the disease, that is, soaring proliferation, apoptosis resistance, necrogenesis, and diffuse invasion. In this paper, we will focus on Bcl-2 family proteins, which emerged as important antiapoptotic proteins limiting the effectiveness of conventional and molecularly targeted therapeutics. We will describe the complex *modi operandi* of canonical and atypical Bcl-2 proteins, discuss current strategies to modulate the activity of critical family members for the treatment of malignant glioma, and conclude with an outlook on future challenges to conclusively translate such basic and translational knowledge into clinical application.

## 2. The Bcl-2 Protein Family—Many White and a Few Black Sheep

**Figure 1:**
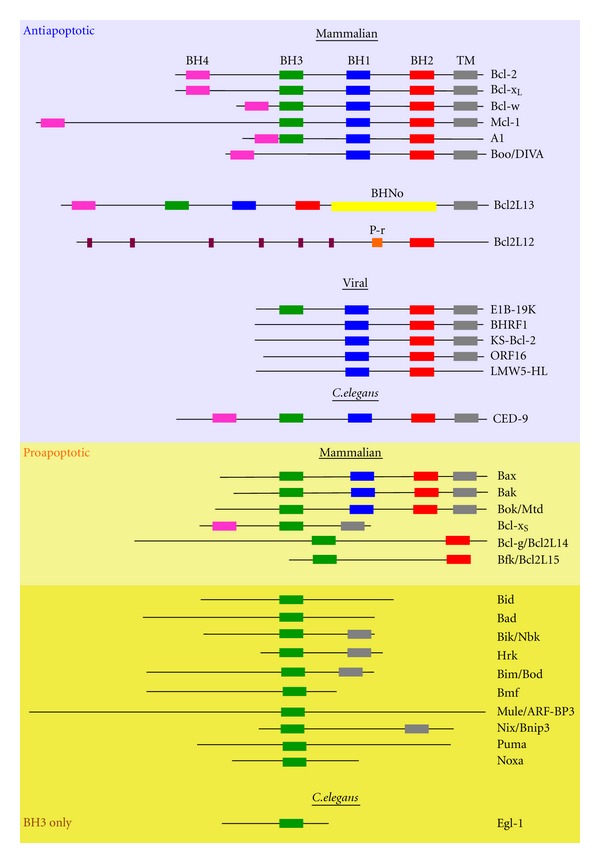
The Bcl-2 family. Domain structure of mammalian, viral, and *C. elegans* proteins. Shown are antiapoptotic “core” proteins, atypical Bcl-2-like proteins, for example, Bcl2L12 and Bcl2L13, proapoptotic multidomain, and BH3-only proteins. Bcl2L12 is characterized by six PxxP motifs located in the N-terminal and center portion of the molecule; these PxxP motifs are depicted as dark red boxes. BH: Bcl-2 homology domain; BHNo: no BH domain; TM: transmembrane domain. Bcl-2: B-cell lymphoma-2; Mcl-1: myeloid cell leukemia sequence 1 (Bcl-2-related); Bim: Bcl-2-interacting mediator of cell death; Bad: Bcl-x_L_/Bcl-2-associated death promoter; Bid: Bcl-2-interacting domain; Puma: p53 upregulated mediator of apoptosis; Bik: Bcl-2-interacting killer; Bmf: Bcl-2-modifying factor; Hrk: Harakiri; Bcl2Lxx: Bcl-2-Like xx; Bok: Bcl-2-related ovarian killer protein; ORF: open reading frame; Bax: Bcl-2-associated X protein; Bak: Bcl-2-antagonist/killer 1; CED-9: cell death abnormality family member; Bnip3: Bcl-2/adenovirus E1B 19 kDa interacting protein 3; Egl-1: egg laying abnormal-1; Mule: Mcl-1 ubiquitin ligase E3. Size of proteins is only approximate.

Apoptosis plays fundamental roles in embryogenesis, immune system homeostasis, and in diverse pathological conditions including cardiovascular, neurological, autoimmune, sepsis-related, and neoplastic disorders [3]. Evading apoptosis by upregulation of antiapoptotic or downregulation of proapoptotic proteins is an important step in gliomagenesis and determines susceptibility to various chemotherapy and radiation modalities. Members of the B-cell CLL/Lymphoma (Bcl)-2 family of proteins as prominent regulators of apoptosis signaling are often misappropriated in many cancers, including lung carcinoma, lymphoma [4–6], and GBM (see below), and consequently emerged as therapeutic targets. Bcl-2 proteins are characterized by the presence of at least one of four Bcl-2 homology domains (amino acid sequence LxxxGD; BH1–BH4). Based on their domain structure and function, the Bcl-2 family is divided into three subfamilies (Figure 1). The first “core” subfamily contains proteins that possess four BH domains and exhibit antiapoptotic functions, such as prototypic Bcl-2 and Bcl-x_L_. Additional members include Bcl-w, A1, Mcl-1, Bcl2L13/Bcl-rambo, Boo/Diva/Bcl-b/Bcl2L10, and Bcl-g. The second subfamily consists of proapoptotic proteins, which contain multiple BH domains, such as Bax, Bak, and Bok. Members of the third subfamily, also referred to as “BH3-only” proteins, lack BH1, BH2, and BH4 cassettes and are characterized by a single BH3 domain. Members of this family are proapoptotic Bid, Bim, Bad, Bik/Blk/Nbk, Mule, Hrk/DP5, Puma/Bbc3, and Noxa (see Figure 1 for schematic representation of mammalian, viral, and *C. elegans* Bcl-2 proteins). 

Over the past two decades, cell culture and animal studies exploited the roles of Bcl-2 family proteins as pro- and antiapoptotic mitochondrial effectors in carcinogenesis and therapy resistance. Bcl-2 family proteins are critical regulators of the intrinsic, that is, mitochondria-dependent apoptotic pathway. Sharply contrasting the extrinsic pathway, which is instigated by death receptor (DR) ligation and activation of initiator caspases 8, 10, and 2 [7], the intrinsic pathway is triggered by chemotherapeutic drugs, developmental cues, and growth deprivation and culminates in the disintegration of inner and outer mitochondrial membranes and the release of apoptogenic factors from mitochondria into the cytoplasm, most notably cytochrome c (cyt c) [3]. Bcl-2 family members regulate mitochondrial membrane homeostasis, by promoting or inhibiting mitochondrial outer membrane permeabilization (MOMP), and consequently the release of proapoptotic mitochondrial factors [8]. Antiapoptotic members, such as Bcl-2 and Bcl-x_L_, are embedded into organelle membranes, most importantly the outer mitochondrial membrane, where they can bind their BH3-only prodeath relatives, such as caspase-activated Bid, termed truncated Bid (tBid). tBid displaces multi-BH domain-containing proapoptotic death agonists, such as Bad and Bax, from a heterodimeric complex with Bcl-2 or Bcl-x_L_. Once released, Bad/Bax oligomerize and insert into the outer mitochondrial membrane to induce MOMP [8]. Mechanistically, a Bax:BH3 alpha-helix interaction triggers conformational changes within the Bax polypeptide, mobilizing a carboxy-terminal alpha helix for membrane translocation and exposing the BH3 domain of Bax, which subsequently interacts with an internal Bax activation site to activate monomeric Bax [9]. Subsequently, mitochondrial proteins, foremost cyt c, are released into the cytosol where they induce caspase activation. By analogy to membrane-bound DRs acting as scaffold proteins to promote caspase maturation, the apoptotic protease-activating factor (Apaf-1) assembles a cytosolic, multimeric structure to enable caspase-9 activation in the presence of cyt c and ATP [10]. Once activated, caspase-9 cleaves downstream caspases, most importantly, effector caspase-3 and -7, which in turn selectively cleave and inactivate cellular proteins to induce and propagate cellular demise. Besides cyt c, second mitochondria-derived activator of caspases (SMAC)/direct inhibitor of apoptosis-binding protein with low pI (DIABLO) and Omi represents a second class of pro-death, apoptogenic factors that are released during MOMP into the cytosolic compartment. They prevent inhibitor of apoptosis proteins (IAPs) from binding and inhibiting postmitochondrial caspases 9, 3, and 7 [11]. 

Bcl-2 was first identified as a deregulated oncogene in follicular B-cell lymphomas, where it is placed under the control of the immunoglobulin heavy-chain promoter by a t(14;18) chromosome translocation. While canonical Bcl-2 proteins play pivotal and well-defined roles in several cancers, including B-cell lymphoma, breast carcinomas, and melanoma, their impact on gliomagenesis is less well understood. During progression from initial to recurrent GBM, the Bcl-2 rheostat was demonstrated to shift towards an antiapoptotic setting, which favored resistance [12]. In a mouse model of oligodendroglioma, coexpression of PDGF-B and Bcl-2 promoted tumor growth and progression to anaplastic disease. Such progression was associated with reduced tumor latencies, increased intratumoral proliferation, and decreased apoptosis rates [13]. Counterintuitively and similar to other malignancies, including mammary and gastric carcinomas, overexpression of antiapoptotic members is more often found in lower-grade tumors than in high-grade gliomas [14] and has repeatedly been associated with a more favorable prognosis [15]. Similarly, high levels of proapoptotic Bax have been observed in advanced gliomas, and to a lesser extent in earlier-stage neoplasms [16]. Although further experimental evidence is required to conclusively address this conundrum, it is conceivable that sustained survival linked to high Bcl-2 expression may reduce the selection pressure to acquire further genetic mutations associated with advanced tumor progression. Another explanation is that Bcl-2 has anti-growth activities, as it delays cell cycle progression through upregulation of the CDK inhibitor, p27, and downregulation of phosphorylated Rb and functional E2F proteins. Accordingly, lymphomas with elevated Bcl-2 levels show lower proliferative capacities, suggesting that the impact of Bcl-2 on G0 to S phase transition is physiologically relevant [17] and that high-grade gliomas may escape cell cycle block by downregulating Bcl-2 expression. Furthermore, antiapoptotic Bcl-2 can induce apoptosis when bound to orphan nuclear receptor Nur77/TR3. Binding of Bcl-2 to Nur77 induces a Bcl-2 conformational change that exposes its BH3 domain, transforming Bcl-2 into a proapoptotic protein [18]. Correspondingly, Bax can act as a potent inhibitor of apoptosis in neurons of mice infected with Sindbis virus [19]. Other studies reporting bimodal activity profiles of Bcl-2 family members identified Bak as a cell-type-specific anti- or proapoptotic protein in neurons depending on neuronal subtype and postnatal development stage [20] and proapoptotic Bid as a pro-survival factor, as its phosphorylation by ATM redirects the cellular program from apoptosis to S phase arrest to allow for DNA repair rather than cell death induction [21, 22]. Finally, Bcl-2 has promigratory and proinvasive activities, as Bcl-2-expressing glioma cell lines exhibited enhanced expression and activity of the proprotein convertase furin, which proteolytically activates proinvasive metalloproteinases (MMP) and TGF-*β*  [*16*]. Further molecular insights into these bimodal activities of Bcl-2 family proteins in glial cells will advance our understanding of the contribution of Bcl-2 family proteins to gliomagenesis. 

In addition, the constitutively active form of EGFR, termed EGFRvIII, resulting from a deletion of exons 2 to 7, inhibited therapy-induced apoptosis via upregulation of Bcl-x_L_ and subsequent blockage of effector caspase activation [23]. In addition, EGFR and EGFRvIII can interact with Puma, resulting in cytoplasmic sequestration and inactivation of Puma [24]. Furthermore, studies in nonglial tumor cells have identified important signaling concepts linking RTK inhibition to apoptosis signal transduction. Erlotinib and gefitinib induced apoptosis in small cell lung cancer cell lines and derived xenografts via upregulation of Bim [25–29]. The Mek-Erk1/2-Notch signaling pathway can induce Bim mRNA and protein synthesis, and consistently, pharmacological inhibition of Mek-1/2 led to upregulation of Bim. Additional studies showed that blockage of the PI3K signaling arm provoked downregulation of Mcl-1, suggesting that the Bcl-2 protein rheostat might determine resistance and sensitivity to inhibition of RTKs and canonical downstream signaling [25, 28, 30]. Similar detailed biochemical and genetic studies analyzing cellular and molecular responses to RTK inhibition are still pending for glioma cells and derived tumors: Is the Bim/Mcl-1 rheostat important for determining sensitivity of GBM tumors to RTK inhibition? Does inhibition of different RTKs converge on Bim upregulation to induce apoptosis or do individual RTKs induce a unique transcriptomic and proteomic signature? Do tumors that are resistant toward RTK inhibitors express antiapoptotic Bcl-2 family members to block Bim-instigated MOMP? Are elevated Bim levels a consequence of transcriptional induction or posttranslational stabilization? Detailed transcriptomic and proteomic surveys of glioma cells and tumors treated with different combinations of inhibitors targeting RTKs and downstream Ras/Mek/Erk and PI3K/Akt/mTOR signaling will provide important answers.

More recently, comprehensive oncogenomic and tissue microarray analyses validated robust amplification (nonfocal gain of chromosome 19q13) and overexpression of the noncanonical Bcl-2 family protein, Bcl-2-Like 12 (Bcl2L12) in primary GBM tumor specimens. In contrast, Bcl2L12 exhibited low or undetectable levels in cells of glial origin in normal brain surrounding tumor tissue or in low-grade astrocytoma [31]. Bcl2L12 lacks BH1, BH3, BH4, and transmembrane motifs and is characterized by a single, carboxy-terminal BH2 domain (Figure 1) [32]. In addition, the Bcl2L12 polypeptide contains a proline-rich region with homology to known oncogenes, such as RRas, and several PxxP tetrapeptide motifs, documenting that Bcl2L12 represents a structurally distinct Bcl2L12 family protein [31, 32]. Does Bcl2L12 impact MOMP similar to canonical Bcl-2 proteins, or does its atypical domain structure translate into atypical functional properties?

## 3. The Black Sheep Bcl2L12: The Bcl-2 Family Meets Postmitochondrial Caspase and Nuclear p53 Signaling

Detailed functional studies in primary and transformed glial cells and derived orthotopic explants identified Bcl2L12 as a potent inhibitor of postmitochondrial effector caspase activation and the p53 tumor suppressor activity. Specifically, enforced expression of Bcl2L12 in primary cortical astrocytes enhanced cellular growth, conferred marked apoptosis resistance toward chemo- and radiation therapies, yet engendered cellular necrosis and effected malignant transformation [31]. In addition, RNAi loss-of-function studies demonstrated that Bcl2L12 neutralization sensitized glioma cells toward apoptosis, and most importantly, reduced intracranial tumor formation with increased apoptotic, decreased proliferative indices, and enhanced tumor-free survival [31]. On the biochemical level, these oncogenic actions related significantly to the capacity of Bcl2L12 to inhibit apoptosis by neutralizing effector caspase-3 and -7 activity downstream of mitochondrial dysfunction and apoptosome activity [31, 33, 34]. Intriguingly, postmitochondrial caspase activation acts as a molecular switch between apoptotic and necrotic cell death: due to mitochondrial dysfunction and cytochrome c release in the context of concomitantly neutralized effector caspase activation, respiratory function and consequently intracellular ATP levels decrease; ion homeostasis can no longer be maintained, provoking cellular edema, dissolution of organelles, and rapture of the plasma membrane [35]. Indeed, Bcl2L12 expression promoted necrosis in response to apoptotic stimulation [31]. In addition, Bcl2L12 physically interacts with and neutralizes the transactivational activity of the p53 tumor suppressor protein by abrogating p53 binding to its target gene promoters. Bcl2L12-mediated neutralization of p53 activity enabled glioma cells to bypass p53-dependent replicative senescence and inhibited DNA damage-induced apoptosis. In addition, Bcl2L12 blocked p53's capacity to bind to certain target promoters and attenuated p53-mediated induction of selected target genes, including the cell cycle inhibitor p21 and the proapoptotic Bcl-2 family proteins Bax, Puma, and Noxa [36, 37]. By blocking apoptosis and p53 signaling and redirecting the death program to necrosis, the molecular profile of Bcl2L12 provides a rational molecular explanation for hallmark features of GBM—that is, apoptosis resistance, florid necrosis, and soaring proliferation—and points to Bcl2L12 upregulation as a key progression event in malignant glioma. 

Recent in-depth analyses of the TCGA (The Cancer Genome Atlas) dataset, a comprehensive catalogue of genomic and genetic aberrations observed in GBM, classified tumor specimens according to their molecular signature into classical, mesenchymal, proneural, and neural subgroups [38, 39]. The Classical group is characterized by increased amplification/expression and mutation of the epidermal growth factor receptor (*EGFR*), deletion of *PTEN* and *INK4A*, and deregulation of retinoblastoma (Rb) pathway components (*RB1*, *CDK4,* and *CCDN2 *genes). The mesenchymal group exhibits loss of the tumor suppressor *NF1* and increased levels of NF-*κ*B signaling molecules, for example, *TRADD*. Elevated expression of neuronal markers, such as *NEFL* and *GABRA1*, mainly characterizes the neural subclass. Finally, proneural tumors show alterations in the *PDGFR, IDH1, *and* TP53 *as well as high expression of oligodendrocytic development genes, such as *PDGFRA*, *NKX2-2,* and *OLIG2*. Importantly, Bcl2L12 exhibited low-level amplification and expression in the proneural class harboring p53 pathway mutations, and higher amplification and expression in EGFR-driven classical and mesenchymal subtypes [36, 37], suggesting that tumors with already incapacitated p53 signaling circumvent the need for additional gain of the p53 inhibitor Bcl2L12, thus providing genetic evidence of a Bcl2L12-p53 axis in GBM. Coexpression of Bcl2L12 and EGFR also raises the possibility that EGFR and Bcl2L12 signaling cooperate to drive gliomagenesis. Future studies will test this provocative hypothesis.

## 4. Targeting the Bcl-2 Family to Block GBM Progression

Several small molecules have been synthesized that act as BH3 mimetics to disarm antiapoptotic Bcl-2 and Bcl-x_L_ by preventing their heterodimerization with, and inactivation of, their proapoptotic relatives. This class of apoptosis inhibitors includes BH3I-1, HA14-1antimycin A, gossypol (NSC19048), apogossypol (NSC736630), obatoclax (GX15-070), chelerythrine, TW-37, 4-(3-methoxyphenylsulfonyl)-7-nitro-benzofurazan-3-oxide (MNB), TM12-06, ABT-737 and the related orally active derivative ABT-263, and pyrogallol-based molecules. While the exact target specificity remains elusive for most of these compounds, many preclinical and clinical studies have documented potent antitumor activities, primarily in lymphoid malignancies. 

Only three Bcl-2 mimetics, HA-14-1, ABT-737, and gossypol have been evaluated as putative antiglioma therapeutics. HA-14-1 and ABT-737, which are the most potent BH3 mimetic with high affinity (*K*_*i*_ ≤ 1) to Bcl-2, Bcl-x_L_, and Bcl-w, induce apoptosis in glioma cell lines by promoting the release of proapoptotic Bax from a Bax:Bcl-2 heterodimeric complex [40, 41]. In addition, ABT-737- and HA-14-1-treated glioma-bearing mice showed reduced tumor formation and increased survival, suggesting that targeting Bcl-2/Bcl-x_L_/Bcl-w alone or in combination with chemotherapy and radiation regimens represents a promising therapeutic approach. Of note, resistance to ABT-737 has mainly been linked to high expression of Mcl-1, suggesting that dual inactivation of Bcl-2/Bcl-x_L_/Bcl-w and Mcl-1 signaling axes is obligatory for substantial apoptosis induction. Expectedly, RNAi-mediated downregulation of Mcl-1 in glioma cell lines and stem cells was found to increase sensitivity to ABT-737-induced apoptosis [40].

While ABT-737 is a highly selective BH3 mimetic, gossypol has activities in addition to binding to the BH3 pocket of antiapoptotic Bcl-2/Bcl-x_L_. These include association or intercalation into plasma and mitochondrial membranes, which results in lipid condensation/damage, gradual mitochondrial swelling, structural distortion of the inner mitochondrial membrane, and dissipation of ΔΨ_*M*_ [42]. Early clinical studies with gossypol revealed little toxicity and low, but measurable response rates in patients with grade III and grade IV astrocytomas [43]. Current clinical trials (NCT00540722, NCT00390403) aim to assess tumor response rates and dosage/toxicity in combination with TMZ/radiation. These trials will provide the first evidence as to whether Bcl-2 protein inhibition mediates antiglioma activity *in vivo*. 

Additional strategies to target Bcl-2 family proteins, not all of which have been tested in malignant glioma, include antisense approaches and diverse compounds such as tetrocarcin A (TC-A), antimycin A3, chelerythrine, and WP1066 [44, 45]. WP1066 selectively inhibits the STAT3 pathway and reduces STAT3-driven expression of Bcl-2, Bcl-x_L_, and Mcl-1, triggering Bax activation [46] and resulting in extended survival of xenogeneic glioma mouse models [47]. Of particular interest are hydrocarbon-stapled, alpha-helical BH3 peptides, which bind with high affinity to multidomain Bcl-2 members, are protease-resistant, and potently induce apoptosis in leukemia cells and derived explants [48]. 

Research over the last five years established microRNAs (miRNAs) as important regulators of gliomagenesis. miRNAs are small, noncoding RNAs that can repress gene expression by either directly binding and inducing degradation of target mRNAs or by inhibiting translation [49]. Specifically, miR-153 has been identified as a negative regulator of Bcl-2 and can induce cell death in GBM [50]. Conversely, overexpression of miR-21 was reported to reduce Bax levels in GBM and protect glioma cell lines from TMZ-induced apoptosis [51]. Therefore, enforced expression of miR-153 or targeting miR-21 with synthetic inhibitors could sensitize glioma cells to therapy-induced apoptosis. 

Additional approaches focused on the overexpression of proapoptotic molecules, for example, Bax and Bik. Adenoviral expression of Bik neutralized Bcl-2 and Bcl-x_L_ and induced p53- and caspase-independent cell death in GBM cell lines. This strategy, however, was limited by significant toxic effects of Bik overexpression to the surrounding glial and neuronal cells [52]. Similarly, Bax overexpression induced apoptosis in glioma cell lines and increased their sensitivity toward radiation therapy [53, 54].

## 5. Inhibitors of the Bcl-2 Protein Family for the Treatment of GBM—Future Perspectives

Antiapoptotic Bcl-2 proteins, for example, prototypic Bcl-2 and Bcl-x_L_, are commonly upregulated in human cancers and counteract the activity of their proapoptotic relatives, which are induced as potent tumor suppressors during cancer progression. Numerous studies evaluated expression levels of canonical Bcl-2 family proteins in hematological malignancies and certain solid neoplasms. In particular, Bcl-2 was shown to be upregulated in chronic lymphocytic leukemia, diffuse large B-cell lymphomas, mantle cell lymphomas, and diverse solid tumors, including neuroblastoma; Bcl-x_L_ overexpression is important for multiple myelomas; Mcl-1 has been implicated in the genesis of acute myeloid leukemia, cholangiocarcinomas, and follicular lymphomas; A1 is expressed in B-cell lymphomas and chronic myeloid leukemia, and high protein abundance of Bcl-w was discovered in advanced-stage colorectal adenocarcinomas [55]. A more detailed expression survey of these Bcl-2 proteins in malignant glioma, in particular the assessment of their inducible expression upon (targeted) drug treatment, remains elusive. 

Given the prominence of antiapoptotic Bcl-2 proteins in driving tumorigenesis and shaping therapy responses, BH3 mimetics emerged as potent antineoplastic therapeutics, either alone or in combination with other modalities, such as DNA-damaging agents. Importantly, ABT-737 and ABT-263 target Bcl-2 and Bcl-x_L_, but not Mcl-1 [56]. Thus, the development of Mcl-1-specific inhibitors remains a high-priority area of investigation. In addition, the elucidation of the precise activation mechanism of proapoptotic Bcl-2 family protein, foremost Bax and Bak, and their transcriptional and posttranslational regulation will most certainly guide the development of targeted agents to specifically activate these proteins. 

Besides small-molecule-based approaches to inactivate antiapoptotic Bcl-2 family proteins, RNAi emerged as a powerful technology, to neutralize expression of Bcl-2, Bcl-x_L_, or Bcl2L12, to sensitize glioma cells toward therapy-induced apoptosis and to reduce intracranial tumor formation in xenogeneic explant models [31, 57]. 

Notably, the systemic delivery of small molecules and RNAs is limited by the low permeability of the BBB/BTB and is often associated with significant cytotoxic effects to non-cancer cells. The recent development of innovative nanotechnological agents to specifically and effectively deliver cytotoxic drugs or small RNA molecules to glioma, but not adjacent normal cells, promises to be transformative for neurooncology research. A recent approach developed by Bhatia and colleagues employed dendrimer-conjugated magnetofluorescent nanoconstructs (called “dendriworms”) as a platform to deliver siRNA *in vivo*. These dendriworms are nontoxic, bypass the BBB, are readily internalized by cells, and can achieve gene knockdown in an acute-onset GBM mouse model *in vivo* [58]. Another recent study by Ding and colleagues delivered antisense oligonucleotides (AONs) targeting laminin-411 to xenogenic glioma explants using a poly (*β*-L-malic acid) nanobioconjugate. This nanomaterial was functionalized with a monoclonal antibody against transferrin receptor (TfR) to facilitate penetration of the BBB and BTB. Intravenous delivery of AON nanoconjugates resulted in potent, glioma-specific knockdown of lamin-411 and, consequently, robust reduction in tumor formation [59]. Future studies will focus on similar delivery systems to neutralize expression of antiapoptotic Bcl-2 family proteins, foremost Bcl-2, Bcl-x_L_, Mcl-1, and Bcl2L12. 

Finally, it will be important to understand the role(s) of Bcl-2 proteins in the maintenance and therapy resistance of glioma stem cells (GSCs), a small cell population embedded within GBM tissue that undergoes self-renewal in culture, forms tumors in orthotopic transplants *in vivo*, can generate a diversified neuron-like and glia-like postmitotic progeny, and is thought to play important roles in GBM recurrence [60]. The key questions are: what are the expression patterns of Bcl-2 family proteins in GSCs? Do Bcl-2 family proteins regulate therapy resistance of GSCs, and if so, what are the underlying molecular mechanisms? A recent study began to address these questions and demonstrated that ATF-5-dependent upregulation of Mcl-1 plays critical roles in GSC survival in response to targeted therapies [61]. 

The molecular characterization of Bcl-2 family continues to establish novel links between Bcl-2 proteins and diverse cellular processes, such as the regulation of mitochondrial energy metabolism and dynamics, glucose metabolism, autophagy, p53 function, calcium signaling, GSC homeostasis, and therapy responses [62]. Such detailed molecular characterization, together with more detailed studies of the role of Bcl-2 family proteins in gliomapathogenesis, foremost the generation of Bcl-2 protein-driven, inducible gain- and loss-function glioma mouse models, and the development of novel (nano-) technologies to effectively deliver therapeutics targeting the Bcl-2 protein family into intracerebral tumors, promise to be transformative for glioma research in the near future.
